# Exposure of Hyperandrogen During Pregnancy Causes Depression- and Anxiety-Like Behaviors, and Reduced Hippocampal Neurogenesis in Rat Offspring

**DOI:** 10.3389/fnins.2019.00436

**Published:** 2019-05-08

**Authors:** Juan Cheng, Haojuan Wu, Huawei Liu, Hua Li, Hua Zhu, Yongmei Zhou, Hongxia Li, Wenming Xu, Jiang Xie

**Affiliations:** ^1^Chengdu Third People’s Hospital, Affiliated Hospital of Southwest JiaoTong University Medical School, Chengdu, China; ^2^Department of Clinical Medicine, Southwest Medical University, Luzhou, China; ^3^Department of Obstetrics and Gynecology, West China Second University Hospital, Key Laboratory of Birth Defects and Related Diseases of Women and Children, Ministry of Education, Sichuan University, Chengdu, China; ^4^Joint Laboratory of Reproductive Medicine, SCU-CUHK, West China Second University Hospital, Sichuan University, Chengdu, China

**Keywords:** androgen, neurogenesis, hippocampus, depression, lithium chloride

## Abstract

The hippocampus is a region in which neurogenesis persists and retains substantial plasticity throughout lifespan. Accumulating evidences indicate an important role of androgens and androgenic signaling in the regulation of offspring hippocampal neurogenesis and the survival of mature or immature neurons and gliocyte. Hyperandrogenic disorders have been associated with depression and anxiety. Previous studies have found that pregnant hyperandrogenism may increase the susceptibility of the offspring to depression or anxiety and lead to abnormal hippocampal neurogenesis in rats. In this study, pregnant rats were given subcutaneous injection of aromatase inhibitor letrozole in order to establish a maternal hyperandrogenic environment for the fetal rats. The lithium chloride (LICl) was used as an intervention agent since a previous study has shown that lithium chloride could promote neurogenesis in the hippocampus. The results revealed that pregnant administration of letrozole resulted in depressive- and anxious-like behaviors in the adolescent period. A remarkable decrease in immature nerve cells marked by doublecortin and mature neurons co-expressed by Brdu and NeuN in adult years were detected in the hippocampal dentate gyrus of adolescent rats. Lithium chloride alleviated the effects on neurobehavioral and promoted the differentiation and proliferation of neural progenitor cells, while a hyperandrogenic intrauterine environment had no effects on astrocytes marked by GFAP in the dentate gyrus. Furthermore, the Wnt/β-catenin signaling pathway related to normal development of hippocampus was examined but there was no significant changes in Wnt signaling pathway members. Our study provides evidence that exposure of androgen during pregnancy leads to alterations in depressive, anxious and stereotypical behaviors and these phenotypes are possibly associated with changes in neurogenesis in the dentate gyrus.

## Introduction

In mammals, sex steroid hormones are synthesized in the gonads: ovary for 17β estradiol (E2) and progesterone (P), and in the Leydig cells of testes for testosterone (T) and have effects on many tissues, including the gonads, the liver, and the nervous system. Such steroids, either via *de novo* synthesis from cholesterol or from local metabolism of steroid intermediate produced in the periphery, can rapidly modulate neuronal excitability and functions, control brain plasticity, and behavior. The *de novo* steroid synthesis occurs in the brains of mammals, called neurosteroids. Neurosteroidogenesis maintains an intense neurogenic activity during adulthood in numerous regions, one of key regions for neurobehavior is hippocampus.

The hippocampus is a region in which neurogenesis persists (Eriksson et al., 1998; Spalding et al., 2013; Woodward et al., 2018) and retains substantial plasticity for the whole life including in humans (Jessberger et al., 2007; Wainwright et al., 2016; Hall et al., 2018). Much research has shown that the compromised hippocampal neurogenesis is attributed to multiple neuropsychiatric diseases, including depression (McKinnon et al., 2009) and dementia (Henneman et al., 2009). Hippocampus neurogenesis also has effect on cognition (Sweatt, 2004) and mood regulation (Campbell and Macqueen, 2004), dysregulation of which is particularly susceptible to depression (Liu et al., 2017). Therefore, the behavior change is often related to the compromised neurogenesis.

Accumulating evidences indicate that androgens and androgenic signaling modulate the hippocampal neurogenesis (Galea et al., 2013). Androgens are the crucial gonadal hormones in men, derived from cholesterol via progestins, including testosterone and androstenedione, which may also be converted to 17β-estradiol via aromatase. Previous evidences show that exposure to androgens for a long time increase hippocampal neurogenesis via modulating the survival of new neurons (Nelson, 2011) within the dentate gyrus (DG), which have been specifically contributed to the activation of the androgen receptor (AR) in rodents, while estradiol has no significant effect on them (Spritzer and Galea, 2007; Carrier and Kabbaj, 2012). A recent study shows that the AR is widely expressed in the developing cortex and hippocampus in mice, and their sexual dimorphism of expression indicates the sex-specific role in behavior regulation (Tsai et al., 2015). Pregnancy and the postpartum period are accompanied with a significant change in steroid levels and peptide hormones, which are necessary for offspring survival (Kinsley and Lambert, 2008). Studies have shown that Wnt plays an important regulatory role in the normal development of the cerebral cortex and hippocampus (Li and Pleasure, 2005; Machon et al., 2007), and can promote the self-renewal and differentiation of prostate cancer cells with stem cell characteristics (Bisson and Prowse, 2009). Wnt signaling in the early stage of neurogenesis plays a role in regulating the self-renewal and survival of neural progenitor cells and inducing the differentiation of neural progenitor cells at later stage. Interestingly, AR can form complexes with β-catenin, a key effector protein of the Wnt/β-catenin signaling pathway, and in prostate tumors β-catenin regulates activation of downstream AR pathways (Lee et al., 2013). Therefore, the dysregulation of androgen production could have a significant impact on neurodevelopment in the offspring.

In this study, we investigated the effects of the prenatal hyperandrogenic environment on neurobehavioral abnormality and hippocampal neurogenesis in offspring. An aromatase inhibitor was used to elevate testosterone since it has been shown that pregnant treatment leads to elevated testosterone level as well autism-like behavior in rat offspring (Xu et al., 2015). In addition, chronic lithium chloride (LICl), a widely prescribed psychological drug, was given to investigate whether it has an alleviating effect on neurobehavioral and neurogenic abnormalities, providing an experimental and theoretical basis for early clinical intervention.

## Materials and Methods

### Animals

This study was carried out according to the recommendations of the Experimental Animal Management and Ethics Committee of West China Second University Hospital. The protocol was approved by the Committee of West China Second University Hospital. Adult Sprague-Dawley (SD, weighing 220–240 g) rats were ordered from the Chengdu Dashuo Experimental Animals Co., Ltd. The animals were housed at 24 ± 1°C, in a 12 h light/dark cycle (light on at 7:00 AM) with full food and water. All animal experiments were performed in accordance with the recommendation of The Guide for the Care and Use of Laboratory Animals of Sichuan University. In order to have timed mating, virgin female rats were individually mated overnight with one or two adult males. Detection of the vaginal plug was named as the day gestation day 0 (G0). Pregnant female rats were randomly assigned into four groups as described below.

### Experimental Groups

The pregnant female rats were divided into four groups: control group (CTL group, *n* = 6), high androgen exposure group (HA group, *n* = 6), lithium chloride treatment group (LICl group, *n* = 6), and high androgen exposure with lithium chloride treatment group (HA+LICL group, *n* = 6). Similarly, after delivery, the offspring were grouped accordingly (CTL group, *n* = 6, male 4, female 2; HA group, *n* = 4, male 2, female 2; LICl group, *n* = 7, male 1, female 6; HA+LICl group, *n* = 4, male 3, female 1). Letrozole (Novartis Pharma Stein AG, Switzerland) at 1 μg/ml was dissolved in 20% ethanol in sesame oil, in accordance with the method established by Moradi-Azani et al. (2011). Pregnant female rats in the HA group and HA+LICl group received subcutaneous injection of letrozole at 1 μg/kg from G0 to G20, while CTL group and LICl group rats received only the vehicle with the same volume at the same time. The LICl group and HA+LICl group treated offspring were given intraperitoneal injection of lithium chloride (Sigma-Aldrich, United States) 2 mmol/kg at postnatal 9 days (from PND 1 to PND 9), while the CTL group and HA group were given intraperitoneal injection of saline solution of the same volume. At PND45-55 (adolescent rats), behavioral tests were examined as following described. After neurobehavioral detection, Brdu (100 mg/kg) was injected intraperitoneally once a day for three consecutive days. At postnatal day 60 (PND60), immunofluorescence staining and western blot were conducted.

### Cesarean Section From Decapitated Rats and Blood Sampling

All the rats’ brains were removed on G21 and blood serum were collected for ELISA analysis. As described by Vaillancourt et al. (1999), an incision is made through the midsection of the abdomen after removed from the brain and pups were quickly delivered from the isolated uterus. The whole operation was conducted under warm conditions to maintain the pups’ body temperature. After delivery, the pups were placed in standard conditions and raised separately until the weaned females and males on the postnatal day 21 (PND21).

### Detection of Testosterone and Estradiol

All blood samples were centrifuged at 3000 rpm for 10 min. ELISA Kit (MSK, Wuhan, China) was used to examine the testosterone and estradiol concentrations in the serum samples of the pregnant rats. The procedure was conducted according to the manufacturer’s instructions. Briefly, the collected condition medium was added to a well coated with testosterone/estradiol polyclonal antibody and then immunosorbented by biotinylated monoclonal anti-rat testosterone/estradiol antibody at 37°C for 2 h. The color development, catalyzed by horseradish peroxidase, was terminated with 2.5 mol/l sulfuric acid, and the absorption was measured at 450 nm. The protein concentration was calculated by comparing the relative absorbance of the samples with the standards. There was less than 10% cross-reactivity with other steroid hormones, and the information was stated in the manual of the company (MSK, Wuhan, China). The *r*-values of standard curves were greater than 0.99 for both assays.

### Neurobehavioral Detection

At PND45-55 (adolescent) behavioral tests were examined as following described.

#### Forced Swimming Test

The depression status of rats was determined by the forced swimming test device (20 cm in diameter, 40 cm in height). Before the trial, the device was filled with water maintained at 25°C, the height of which meant that rats could not reach the bottom or the upper edge of the cylinder to escape. The camera recorded for 10 min including the time of immobility whenever rats aborted all active behaviors and spent for strongly swimming. After each experiment, the rats should be kept dry on a heater before making their way back to cages. The device was cleaned before next animal.

#### Elevated Plus Maze

The animals’ anxious-like behaviors were detected using the elevated cross maze experimental device, which had two open arms and two closed arms (two arms with 20 cm high walls on both sides, a length of 50 cm). There was a connected platform (10 × 10 cm) in the middle. The rats were placed on the intermediate platform of the device, and the cameras were used to record 10 min, including the time and times of their entering the open arms.

#### Three-Chamber Sociability Test

The three-chamber device was used to test the social communication ability of SD rats. The apparatus consisted of three Plexiglas chambers (40 cm × 20 cm × 20 cm) with each of the side chambers connected to the middle chamber by a corridor (10 cm × 10 cm × 15 cm). One side of the compartment was set up with unfamiliar rats of the same sex and age which had no previous contact, while the other side was set up with a toy. At the beginning of the test, the rat was placed into the middle chamber and allowed the exploration of the three chambers for 5 min. Then a model rat, locked in a small cage, was placed in one of the side chambers and a toy was placed in the other side chamber. The testing rat was allowed to freely explore the apparatus and interact with the model rat for 10 min. The activity of rats in the set was recorded, and the time of communication with unfamiliar rats and the object was counted. All behavioral experiments were carried out during the dark period of the light cycle under dim red illumination.

#### Self-Grooming Test

The open-field experiment device (60 × 60 cm) was used to detect the rats’ repetitive or stereotypical self-combing behavior. Before the test, the rats were placed in the device for 5 min, and the rats were put into the device on the day of the experiment. The activity of the rats was recorded for 15 min and their self-grooming behaviors were counted. Before the next experimental rat, 75% ethanol was used to clean the facility.

### Immunofluorescence Staining

After neurobehavioral evaluation, Brdu (100 mg/kg) was injected intraperitoneally once a day for three consecutive days. At postnatal day 60 (PND60), rats were deeply anesthetized with chloral hydrate. After anesthesia, rats were perfused pericardially with saline and 4% paraformaldehyde buffer. Then, the removed brain tissues were fixed in 4% paraformaldehyde for 48 h, next dehydrated with 10, 20, and 30% sucrose consecutively for 24 h, respectively. After treatment with sucrose solution, the tissues were embedded in paraffin. 30 μm paraffinic cross sections were prepared and examined, and each group were 10 brain slices at the same level. All sections were permeabilized with 0.5% Triton X-100, then blocked with 5% bovine serum albumin (BSA) and incubated with anti-Brdu and NeuN antibody (1:1000; Cell Signaling) mix diluents, anti-Brdu and GFAP (1:1000; Cell Signaling) mix diluents or anti-doublecortin antibody (1:1000; Cell Signaling) overnight at 4°C. Secondary antibodies (1:1000, Thermo Fisher Scientific) were added for 1 h and then incubated with 4,6-diamidino-2-phenylindole (DAPI, Sigma-Aldrich) to label the nuclei, respectively at RT. Finally, confocal microscope (Olympus) was used to acquire the images. At least six sections were used for staining of each rat, and the sequential sections were used in each condition.

### Western Blotting

#### Hippocampus Collection

Approximately at postnatal day 60 (PND60), the rats’ brains of each group were rapidly removed from the skull and dissected brain tissue on ice. At least three rat tissues were extracted in every group. The whole hippocampus was quickly dissected and stored at -80°C for further protein extraction.

#### Protein Extraction

The hippocampal tissue samples were weighed (30 mg) and placed onto a filter cartridge. The tissue was then grounded using the plastic rod for 1–2 min. 100 μl buffer A was added to the filter and mixed well, placing the filter with cap open on ice for 5 min. Next, the proteins of the nuclei, cytosol and plasma membrane were extracted according to the instructions of Minute^TM^ Plasma Membrane Protein Isolation and Cell Fractionation Kit (Beyotime Institute of Biotechnology). The concentration of protein was detected by the BCA Protein Assay Kit (Beyotime Institute of Biotechnology).

#### Western Blotting

The proteins were adjusted to the same concentration with RIPA lysis buffer and added 5× sample loading buffer. 40 μg total protein was separated on a 10% SDS-polyacrylamide gel (SDS-PAGE) and transferred to polyvinylidene difluoride (PVDF) membranes (Millipore, MA). After blocking in 5% BSA for 1 h at RT, the membranes were incubated overnight at 4°C with the following antibodies: anti-GAPDH (1:5000, Zen Bioscience, China), anti-β-catenin (1:400, Abcam, United States), anti-GSK-3β (1:500, Abcam, United States), anti-DVL2 (1:500, Bioss, China), anti-TCF-4 (1:500, Bioss, China). β-catenin, GSK-3β, DVL2, TCF-4 are the main members of Wnt/β-catenin signaling pathway. On the next day, the membranes were washed by Tris-buffered saline Tween-20 (TBST) (3 × 5 min), then incubated with anti-mouse or rabbit secondary antibodies for 1 h at RT. Protein bands on the membrane were detected using an chemiluminescence detection system (Millipore Corporation, United States), following the manufacturer’s instructions. The relative densities of each band were measured using ImageJ (Schneider et al., 2012). All the antibodies were either validated by other studies or by ourselves in a preliminary experiment.

### Statistics

The results were analyzed by SPSS version 19.0 and GraphPad Prism version 5.0. Data were presented as mean ± SEM and comparisons among groups used one-way analysis of variance (ANOVA). For all tests, it is considered as statistically significant for a probability (*p*) value below 0.05 (two-tailed).

## Results

### Serum Testosterone and Estradiol Levels in Pregnant Rats

**FIGURE 1:**
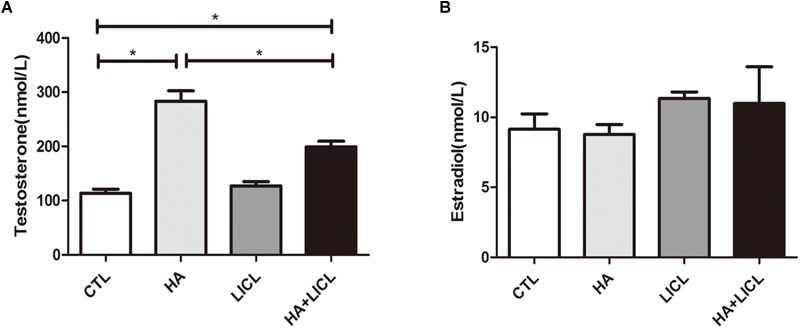
Higher levels of serum testosterone in pregnant rats after letrozole treatment. **(A)** Pregnant rats in the HA and HA+LICl groups (*n* = 6) had higher levels of serum testosterone as compared to the CTL group (*n* = 6), **(B)** There was no significant difference in serum estradiol levels among the four groups. ^∗^*p* < 0.05.

Serum samples from blood of the pregnant rats were collected on G21 before the Cesarean sections. ELISA measurement shows that serum testosterone levels of the pregnant rats were higher in the HA and HA+LICL groups (mean ± SEM: 283.65 ± 46.05 nmol/L, 199.04 ± 10.52 nmol/L, respectively), as compared to the CTL group (113.36 ± 19.39 nmol/L, *p* < 0.05) and LICL group (127.07 ± 13.74 nmol/L, *p* < 0.05). No significant difference was found in serum estradiol levels between the four groups (Figure 1).

### Behavioral Detection of the Offspring

**FIGURE 2:**
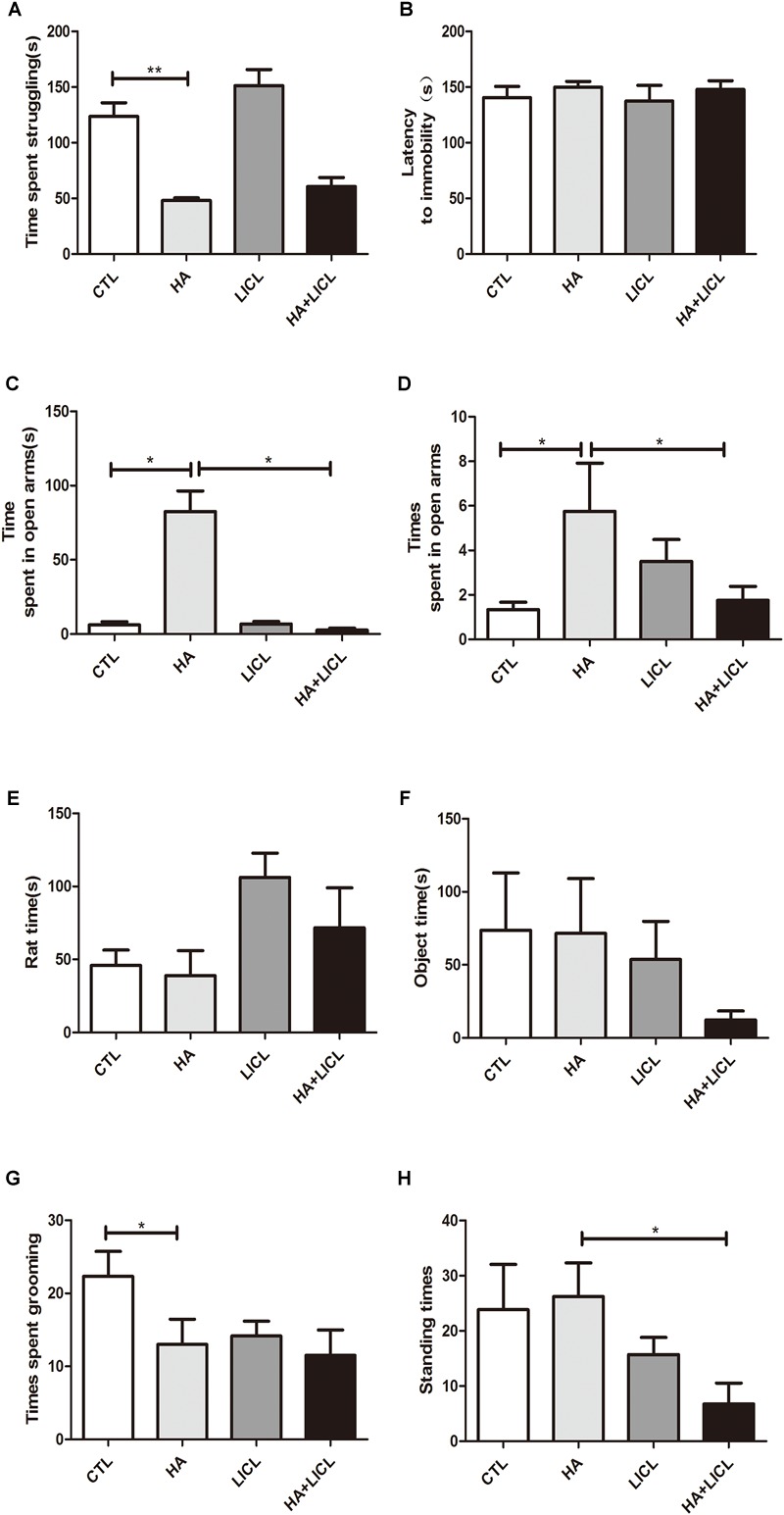
Behavioral test of the offspring. **(A)** Time spent in struggling was significantly decreased after HA treatment **(B)** No significant difference was found on the time of latency to immobility in treatment group. **(C,D)** Both the time and times of their entering the open arms were higher in the HA group as compared to the CTL group and lower in the HA+LICl group as compared to the HA group. **(E,F)** The time of communication with unfamiliar rats and object showed no significant difference following letrozole and lithium chloride treatment. **(G)** The stereotyped behavior of grooming were decreased, **(H)** and the standing behavior were not improved after lithium chloride intervention. ^∗^*p* < 0.05, ^∗∗^*p* < 0.01.

The behavior test was examined in the offspring, and the results show that the time spent in struggling significantly decreased in HA group (38.38 ± 9.36 s) compared to CTL group (123.58 ± 30.39 s, *p* < 0.01) (Figure 2A). No significant difference was found in the time of latency to immobility between the four groups (Figure 2B). Both the time and times for them to enter the open arms were higher in the HA group as compared to the CTL group (*p* < 0.05) (Figure 2C), and lower in the HA+LICL group as compared to the HA group (Figure 2D). The time of communication with unfamiliar rats and object had no significant difference between the four groups (Figure 2E,F). The times spent in grooming were less in the HA group as compared to the CTL group (Figure 2G), and the times spent in standing were lower in the HA+LICL group as compared to the HA group (*p* < 0.05) (Figure 2H). The results show that HA treatment could alter some parameters of behavior and lithium chloride could rescue the phenotypes related to depression and anxiety.

### The Neurogenesis of DG Area

**FIGURE 3:**
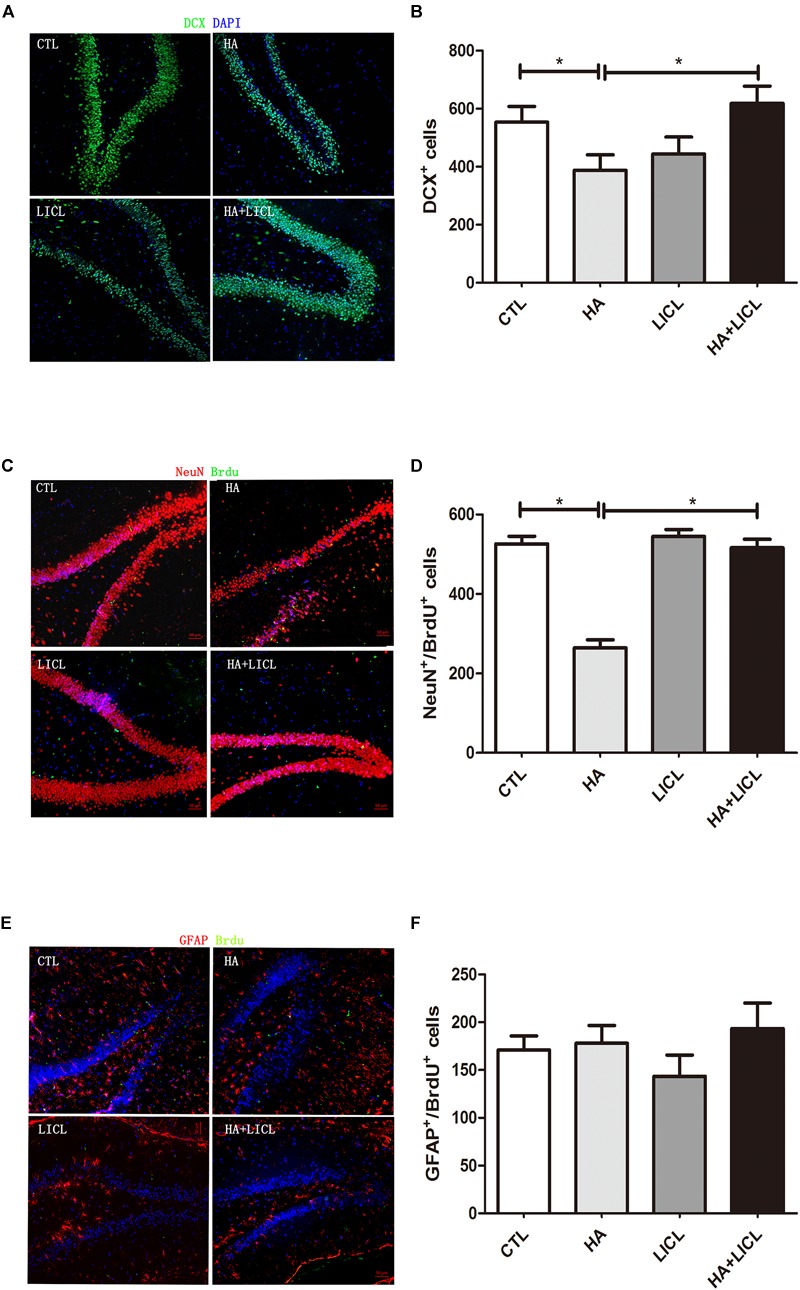
The neurogenesis of DG area among different treatment groups. **(A,B)** The number of DCX positive neurons of the rat DG area was significantly decreased in HA group as compared to CTL group, and after lithium chloride treatment the number of DCX positive neurons increased in HA+LICl group. **(C,D)** Brdu and NeuN neurons in the DG region were reduced, while lithium chloride promoted the proliferation of NPCS in HA+LICl group. **(E,F)** No significant difference was found in Brdu and GFAP neurons among the four groups. ^∗^*p* < 0.05.

The rat DG area Doublecortin (DCX) positive expression of immature neurons in HA group (*n* = 6, 387 ± 53) was significantly decreased compared to CTL group (*n* = 6, 553 ± 54, *p* < 0.05), and after lithium chloride treatment, the number of DCX positive cells increased significantly between HA and HA+LICL group (*n* = 6, 618 ± 59, *p* < 0.05) (Figure 3A,B). In the HA group (*n* = 6, 264 ± 20), both Brdu and NeuN positive neurons in the DG region were more reduced than in the CTL group (*n* = 6,526 ± 19, *p* < 0.05), whereas, after LICl treatment, Brdu and NeuN positive cells increased in HA+LICL group (*n* = 6, 516 ± 21, *p* < 0.05) (Figure 3C,D). There was no significant difference in Brdu and GFAP positive neurons among the four groups (*p*> 0.05) (Figure 3E,F).

### Western Blotting of Wnt/β-Catenin Signaling Pathway

**FIGURE 4:**
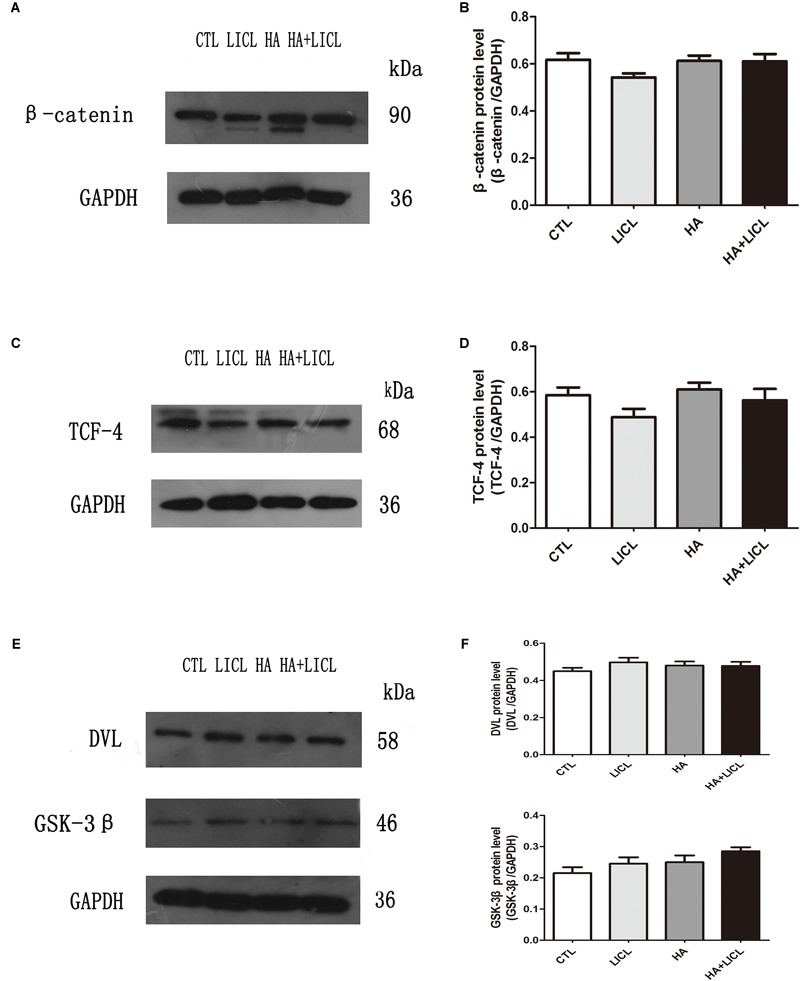
The expression of Wnt/β-catenin signaling pathway proteins. **(A–F)** After letrozole exposure there was no significant difference of β-catenin, TCF-4, GSK-3β and DVL2 between CTL group and HA group. Furthermore, single lithium chloride or co-letrozole treatment had no effects on the expression of four proteins in LICl group and HA+LICl group.

The β-catenin protein was detected in the cytoplasm, TCF-4 detected in nucleus, and GSK-3β and DVL2 in the cell membrane. The results show that there was no significant difference after letrozole or LICl treatment (Figure 4A–F).

## Discussion

The current study shows that after exposure of the mother to hyperandrogenic environment, the offspring presented more depressive- and anxious-like behaviors. This is consistent with previous studies in animal models (Meng et al., 2011; Hernández and Fernández-Guasti, 2018).

Many studies have already shown that chronic stress produces decreased grooming activities (Yalcin et al., 2007), meanwhile in chronic social crowding and isolation stress (Pan et al., 2006; Dronjak et al., 2007). In line with these observations, our studies also indicate the reduced grooming behavior in depression or anxiety condition in our model. Clinical researches have suggested that testosterone administration may alleviate the social anxiety symptoms (Terburg et al., 2016). Prenatal intrauterine exposure to the hyperandrogenic environment of female rats resulted in less time of adolescent offspring in social interaction (Xu et al., 2015). In terms of the impact of androgen on the neurogenesis, one previous study is also controversial as it shows that androgens increase survival of neurons in the dentate gyrus through AR dependent mechanism in male rats (Hamson et al., 2013), while our results indicate the opposite effects in the same region of offspring. Furthermore, our results show that exposure to a maternal hyperandrogenic environment, induced by letrozole, may contribute to neurobehavioral abnormalities, and lithium chloride could rescue the neurogenesis defects in our rat model. It has been shown that the neurogenesis of the hippocampus is compromised in post-mortem tissue of patients accompanied with depression (Boldrini et al., 2012) and Alzheimer’s disease (Crews et al., 2010), as well as in animal models (Mu and Gage, 2011; Wainwright et al., 2011). Therefore, the prenatal hyperandrogenic environment should be regarded as an important etiological factor in neurodevelopmental diseases. Furthermore, studies on patients of Polycystic Ovary Syndrome and Congenital Adrenal Hyperplasia show that they are often more likely to be accompanied with neurobehavior abnormalities (Hamson et al., 2013). Therefore, the study in the current research has broad clinical implications.

One of key findings from the current study is that the reduction of neurogenesis caused by exposure to high testosterone can be restored to a normal level with lithium chloride, supporting an important role of lithium chloride effect in different conditions of neurogenesis defects (Bianchi et al., 2010; Kitamura et al., 2011; Qi et al., 2017; Lewin et al., 2018; Zhang et al., 2018). Lithium chloride has been used as a mood stabilizer for a long time as a clinical treatment of bipolar disorder with mania and depression and for prevention of their recurrence (Manji et al., 2000). In animal models, lithium chloride has therapeutic effects on neurodevelopmental diseases such as Down syndrome (Bianchi et al., 2010) and neurodegenerative diseases such as Alzheimer’s disease (AD) (De Ferrari et al., 2003), as it has the function of protecting and nourishing nerves. It may play a role in promoting the proliferation and differentiation of neural progenitor cells, reducing the apoptosis of neurons and up-regulating the level of neurotrophic factors (Chen et al., 2000; Son et al., 2003). Neurogenesis refers to the proliferation and division of neural stem cells into differentiating progenitor cells, which become mature neurons when migrating to functional areas and establish synaptic connections with other neurons to support neurogenesis (Vogel, 2013; Paridaen and Huttner, 2014). Doublecortin (DCX) functions in the stabilizing microtubules in early mitotic neurons as a marker of immature neurons and is often used to evaluate immature neuronal morphology (Bechstedt et al., 2014), which is expressed in the hippocampus of rat from P0 to P21 after the birth (Brown et al., 2003). NeuN expressed in most mature neurons functions in RNA splicing at the nucleus (Kim et al., 2009; Duan et al., 2016), which is not expressed in certain neuronal populations such as the cerebellar purkinje cells (Mullen et al., 1992). Previous studies have confirmed that neuropsychiatric diseases are often associated with neurological damage of different degrees (Ruksee et al., 2014; Zhang et al., 2014). The current result shows that maternal rats exposed to the hyperandrogenic environment were found to have a reduced number of immature nerve cells marked by DCX and mature neurons co-expressed by Brdu and NeuN during their adult years. Meanwhile, lithium chloride promoted the proliferation and differentiation of neural progenitor cells. The effects of lithium chloride on the neurogenesis support its potential role in neurobehavior intervention.

Letrozole, an aromatase inhibitor was used to elevate testosterone in the current study. Letrozole decreases estrogen expression levels and accumulates androgen in the body (Haynes et al., 2003). This is similar to the results of hyperandrogen, except without any apparent effect on the level of estrogen (Xu et al., 2015), although it contradicts previous research showing a lower concentration after letrozole treatment (Rashid et al., 2015).

The exact pathway of how androgen exposure reduce neurogenesis is not clear, as studies have reported that glycogen synthase kinase 3β (GSK-3β) activation leads to neurogenic damage (Chao et al., 2014; Trazzi et al., 2014). It has also been shown that lithium chloride promoted the proliferation and differentiation of neural progenitor cells through regulating Wnt pathway. However, in the current study, we have detected Wnt signaling molecules using Western blot and found no significant change of Wnt signal molecules. Although the current result did not find any significant change in Wnt molecules after the treatment in the hippocampus, a subtle change in the Wnt pathway, such as β-catenin phosphorylation, cannot be ruled out since more sensitive methods are needed to clarify the issue. In this regard, further experimental studies are needed to explore the underlying mechanism of defective neurogenesis caused by exposure to hyperandrogen during pregnancy and the alleviation of lithium chloride intervention.

## Conclusion

The present study demonstrated that pregnant administration of letrozole resulted in depressive and anxious -like behaviors of rats in the adolescent period. A remarkable decrease in immature nerve cells marked by DCX and mature neurons co-expressed by Brdu and NeuN during adult years were detected in the hippocampus of rats, which indicated that the hyperandrogenic intrauterine environment could induce depression and abnormal hippocampal neurogenesis in rats, and lithium chloride alleviated the effects on neurobehavioral and hippocampal abnormalities. Wnt/β-catenin signaling pathway seems not to be related to this regulation process.

## Ethics Statement

This study was carried out in accordance with the recommendations of the Experimental Animal Management and Ethics Committee of West China Second University Hospital. The protocol was approved by the Experimental Animal Management and Ethics Committee of West China Second University Hospital.

## Author Contributions

JX and WX involved in research design and data interpretation. JC performed the majority of the experimental work and analysis of data, and contributed to writing of the manuscript. HW, HuawL, HuaL, HZ, YZ, and HoL were responsible for the experimental progress.

## Conflict of Interest Statement

The authors declare that the research was conducted in the absence of any commercial or financial relationships that could be construed as a potential conflict of interest.

## Abbreviations

AR: androgen receptor
BSA: bovine serum albumin
DCX: doublecortin
DG: dentate gyrus
E2: estradiol
GSK-3β: glycogen synthase kinase 3β
LICl: lithium chloride
P: progesterone
SDS-PAGE: sodium dodecyl sulfate-polyacrylamide gel electrophoresis
T: testosterone
TBST: Tris-buffered saline Tween-20
